# A Virtual Reality Platform for Evaluating Deficits in Executive Functions in Deaf and Hard of Hearing Children—Relation to Daily Function and to Quality of Life

**DOI:** 10.3390/children11091123

**Published:** 2024-09-13

**Authors:** Shaima Hamed-Daher, Naomi Josman, Evelyne Klinger, Batya Engel-Yeger

**Affiliations:** 1 Department of Special Education, Oranim Academic College, Tiv’on 3600600, Israel; daher.shaima@oranim.ac.il; 2 Department of Early Childhood Education, Beit Berl Academic College, Kfar Sava 4490500, Israel; 3 Department of Occupational Therapy, Faculty of Social Welfare & Health Sciences, University of Haifa, Haifa 3490002, Israel; njosman@univ.haifa.ac.il; 4 Federative Institute for Research on Handicap, University of Bordeaux, 33405 Bordeaux, France; evelyne.klinger@orange.fr

**Keywords:** deaf and hard of hearing children, executive functions, virtual reality, daily activity, quality of life

## Abstract

**Background:** Childhood hearing loss is a common chronic condition that may have a broad impact on children’s communication and motor and cognitive development, resulting in functional challenges and decreased quality of life (QoL). **Objectives:** This pilot study aimed to compare executive functions (EFs) as expressed in daily life and QoL between deaf and hard-of-hearing (D/HH) children and children with typical hearing. Furthermore, we examined the relationship between EFs and QoL in D/HH children. **Methods:** The participants were 76 children aged 7–11 yr: 38 D/HH and 38 with typical hearing. Parents completed the Behavior Rating Inventory of Executive Function (BRIEF) and Pediatric Quality of Life Inventory (PedsQL), while the child performed a shopping task in the virtual action planning supermarket (VAP-S) to reflect the use of EFs in daily activity. **Results:** D/HH children showed significantly poorer EFs (as measured by BRIEF and VAP-S) and reduced QoL. Difficulties in EFs were correlated with lower QoL. BRIEF scores were significant predictors of QoL domains. **Conclusions:** Difficulties in EFs may characterize children with D/HH and reduce their QoL. Therefore, EFs should be screened and treated. VAP-S and BRIEF are feasible tools for evaluating EFs that reflect children’s challenges due to EF difficulties in real-life contexts.

## 1. Introduction

Hearing loss is one of the most common chronic conditions in children, impacting about 34 million children worldwide [1]. Hearing loss encompasses both complete and partial loss of hearing ability [2]. Hearing loss is commonly classified as mild, moderate, severe, or profound [3]. In the United States, the majority of hearing loss cases (50–60%) are genetic, including syndromic (15–30%) and non-syndromic (70–85%) varieties [4,5]. Of the remainder, about 35% of cases are associated with infectious disorders or occur as a result of neonatal events [6]. In addition, the etiology of hearing loss is divided into conductive (due to conditions affecting the external or middle ear), sensorineural (caused by damage or diseases related to the inner ear, such as the cochlea with or without auditory nerve involvement), and mixed hearing loss causes [3]. Hearing loss can affect one (unilateral) or both ears (bilateral). In all cases, hearing loss may lead to functional difficulties and delays in children’s communication, motor, and cognitive development [3,7,8]. Therefore, the World Health Organization (WHO) highlights the necessity of early identification and appropriate intervention measures to ensure children’s optimal development and well-being [9].

When referring to the implications of hearing loss on children’s cognitive abilities, a large body of knowledge emphasizes that the difficulty in adequately perceiving auditory sensory information due to hearing loss may affect children’s language development, verbal memory, attention, working memory, and behavioral regulation [10,11,12,13,14,15]. Working memory and behavioral regulation are components of high cognitive abilities called executive functions.

Executive functions (EFs) refer to complex cognitive processes including control, supervisory, and self-regulatory mechanisms. EFs are responsible for organizing and directing all cognitive activities, emotional reactions, and obvious behaviors. EFs are essential for decision-making and performing meaningful, goal-directed actions [16,17]. Therefore, EFs include capacities such as inhibitory control, selection of relevant task goals, working memory activation, planning and organizing information, solving complex problems, shifting, monitoring a course of action, and evaluating success [16,18]. Because EFs are critical for performing and managing almost all activities of daily life and for adaptive responses to environmental demands [16,19], difficulties in EFs may significantly affect each aspect of daily function. EF difficulties become more visible when an individual is asked to function in a new and unexpected situation when performing multiple tasks simultaneously or new patterns of action [18,20,21]. In children, adequate EFs are a crucial part of their development and are essential for academic achievement, proper social interactions, and daily activity performance [22,23].

Hearing loss during childhood may affect EFs [24,25,26]. Several studies have been conducted on the development of EFs in deaf and hard-of-hearing (D/HH) school-aged children [26,27,28,29,30,31,32,33]. The combined outcomes of these studies show that D/HH children perform significantly lower in all EF tasks compared to their typical hearing peers. Working memory and inhibition are two areas in which differences have been consistently documented [11].

Some authors have explained EF differences by recognizing the complex interrelationships between hearing, language, and EFs [28,30,34,35,36,37]. While some authors have suggested that hearing deprivation has a direct impact on EFs and other higher cognitive processes [34,35], others argue that language deprivation can explain the effect of hearing deprivation on EFs [28,30,36,37]. According to the first hypothesis, hearing loss causes an abnormal bias in developmental and neural connectivity, which may impact EFs and other cognitive processes [34]. The second hypothesis argues that the differences in EFs are caused by a deficiency in language development, given that both are closely related and that language is part of cognitive abilities [37]. A third possibility, proposed by Conway et al. [38], suggests that differences in social environments may impact EFs. Additional studies claim that EF difficulties may be a result of vestibular disorders [39,40], which often present in D/HH children [41]. The relationship between vestibular dysfunction and cognitive impairment remains unclear; however, existing research suggests a potential pathway. The increased gaze and postural instability associated with vestibular loss might demand elevated attentional resources for maintaining balance, consequently reducing the cognitive capacity available for other tasks [39]. Hence, there is no scientific consensus regarding the causal nature of decreased EFs in deaf children. Therefore, more scientific research is needed to elucidate the causes or factors associated with poor EFs in deaf children, allowing us to intervene in these differences immediately and successfully.

Difficulties in EFs among D/HH children may reduce their development, social relationships, and emotional status and can explain variance in learning performance and academic achievement [42,43,44,45,46,47,48,49]. For example, Taljaard et al. indicate that lower hearing levels are associated with lower performance across all cognitive domains, including executive functioning [45]. These concerns have long-term implications for educational and occupational development. For example, Qi and Mitchell [44] found that D/HH children performed consistently poorer in reading and mathematics than typical hearing children. Thus, it is essential to screen for executive dysfunction as early as possible, mainly in vulnerable groups such as D/HH children, to ensure optimal development and function [7,23,31,37].

Most existing studies on EFs in D/HH children examine separate EF components, such as inhibition, shifting, and working memory [11,50]. Data are mainly gathered by neuropsychological measures and in laboratory/clinical settings [24,27,51,52], and lack a comprehensive perspective on the implications of EFs on children’s daily lives. Ecological valid measures that imitate activities in real-life contexts [16,53,54,55] may provide knowledge about how EFs affect the daily functioning of D/HH children. This is in line with the International Classification of Functioning, Disability, and Health (ICF) of the World Health Organization [9], which stresses the necessity of health professionals to consider the implications of a health condition (such as hearing loss) and related body dysfunctions (as EF difficulties) on the individuals’ ability to carry out everyday activities and participate in daily life contexts (home, school, community).

One standardized evaluation that measures how various executive function (EFs) components affect children’s daily lives is the “Behavior Rating Inventory of Executive Function” (BRIEF) [56]. The parents’ version of the BRIEF enables gathering information from the children’s main caregivers, which aligns with the family-centered approach that encourages gathering information from parents to increase their awareness of the challenges and functional limitations their child encounters. However, there is a need to gather information directly from the child [57]. The use of Virtual Reality (VR) to evaluate EFs in actual life directly from the child, in a friendly manner, could provide a solution. VR is a human–computer interaction platform that allows the creation of realistic temporal and spatial situations or objects by simulating real-world conditions [58]. VR as an ecologically valid EF assessment enables physicians to observe their clients in everyday situations while performing a task in real-time, potentially incorporating conventional neuropsychological evaluation methods and enhancing reliability and psychometric validity [58,59].

In the rehabilitative context, VR offers several beneficial qualities, such as the ability to administer instructions and stimuli through multiple senses (auditory, visual, and tactile), which can be tailored to patients’ potential sensory impairments [58]. VR also provides game-like elements that are perceived as interesting and enjoyable by children, enhancing their sense of immersion, motivation, and cooperation [60,61,62]. Additionally, VR enables individualized treatment based on the abilities and needs of the patients, and has been found to increase independent functioning in real-life scenarios for various clinical populations [54,63,64,65]. One of the VR platforms used to evaluate executive function (EF) during instrumental activities of daily living (IADL) is the Virtual Action Planning Supermarket (VAP-S) [66,67]. Shopping activities involve the intense use of EFs, such as planning, working memory, and monitoring, making them a relevant task for assessment [53,68]. The VAP-S simulates a medium-sized supermarket with numerous aisles and records the various EFs used by individuals to successfully purchase a list of daily products [66,69]. Previous studies have demonstrated the VAP-S to be a reliable and valid tool for evaluating EF difficulties in neurological and psychiatric populations [54,70,71,72], with the ability to classify over 70% of participants based on their group and diagnosis [73]. The VAP-S has also shown concurrent validity with other EF assessments, such as the Behavioral Assessment of the Dysexecutive Syndrome (BADS) [74]. However, to the best of our knowledge, this is the first study to apply the VAP-S to children who are deaf or hard of hearing (D/HH).

The present study discusses the importance of quality of life (QoL) in children, particularly for those with chronic conditions such as hearing loss. QoL is defined as an individual’s subjective perception of their physical, social, and psychological functioning [9]. Health-Related Quality of Life (HRQoL) is a subcategory that addresses how a chronic condition affects a person’s QoL [75]. Therefore, it is a valuable method of assessing psychosocial functioning in children with chronic conditions that impact several aspects of their lives, such as hearing loss. Hearing loss has been reported to affect a child’s quality of life [76]. Evidence from previous studies suggests that the consequences of hearing loss extend far beyond audition, negatively affecting components of QoL, such as academic performance [77], emotion and behavior [78], social functioning [79], and psychosocial functioning [80]. Previous studies have found mixed results regarding the impact of hearing loss on children’s QoL, with some studies reporting lower QoL [81,82], while others found no significant differences [83,84,85] or differences only in specific subdomains [80,86]. The inconsistent outcomes may be due to the varying effects of family, peers, and school on children’s QoL, as well as their limited capacity to make significant changes to their environment independently [87]. Consequently, special generic tools for measuring QoL in children have been developed, such as the Pediatric Quality of Life Inventory (PedsQL) [88], which evaluates children’s QoL from both their parents’ and their own perspectives. Since all QoL domains are among those that may be negatively influenced by difficulties in EF, it seems reasonable that children with disorders involving difficulties in EF may be at an increased risk of poor QoL. The most convincing evidence for a link between EF and QoL is provided by conditions such as head injuries and attention-deficit/hyperactivity disorder (ADHD), both of which are related to executive difficulties as primary symptoms [89,90]. Research among these populations indicates that they are more likely to have poor QoL in various areas, such as physical and social functioning, parental emotional well-being, and family related activities [91,92,93]. Furthermore, additional studies have shown that EF can predict QoL in various populations, including pediatric epilepsy [94], diabetes mellitus [95], and children with congenital heart disease [96].

Since hearing loss and EF may affect the child’s physical and psychological health, social interactions, and level of independence, it is important to refer to the interactions between these factors. The present pilot study explored the relationship between EF and QoL among D/HH children. Elaborating knowledge about these interactions may assist in optimizing intervention programs for D/HH children that are focused on improving daily function and QoL.

The present study aimed to (1) compare EF as expressed in daily life (based on parents’ reports, using the BRIEF, and in daily activity performance using the VAP-S) and QoL between D/HH and typical hearing children; (2) examine the relationship between VAP-S and BRIEF scores among D/HH children; and (3) examine the relationship between EF and QoL among D/HH children and the ability of EF to predict QoL domains.

Four hypotheses were posited:

**Hypothesis** **1.** *Differences in EF and QoL are expected between D/HH children and their typical hearing peers.*

**Hypothesis** **2.** *Among D/HH children, deficits in EF as measured by the BRIEF are expected to correlate with performance on the VAP-S.*

**Hypothesis** **3.** *Among D/HH children, deficits in EF as measured by the BRIEF and VAP-S are expected to correlate with QoL.*

**Hypothesis** **4.** *Among D/HH children, EF components would predict various QoL domains.*

## 2. Materials and Methods

### 2.1. Participants

A total of 76 children aged 7–11 years participated in this pilot study: 38 D/HH and 38 typical hearing children. All participants were recruited from regular education schools north of (BLINDED). Based on assessments performed in their school, a normal cognitive level was found in both groups. In the study group, the severity of hearing loss was determined based on audiologic evaluations and medical records found in the school. Participants’ socioeconomic level ranged from low to high based on their parents’ reports about their mean monthly income level (according to the criteria published by the Central Bureau for Statistics in Israel, 2020) [97]. Although the groups were supposed to be matched by sociodemographic parameters, according to the final sample, differences were found in mothers’ years of education and socioeconomic level, which were higher in the normal group, as shown in Table 1.

The exclusion criteria for all participants were as follows: severe/chronic physical/mental health conditions (such as cerebral palsy or intellectual developmental deficits). Deficits were identified based on medical records and parents’ reports. Furthermore, the inclusion criteria for the typical hearing control group were normal hearing (a normal hearing test where the threshold is lower than 20 dB), as reported by parents in the demographic questionnaire.

### 2.2. Measures

#### 2.2.1. A Socio-Demographic Questionnaire

This questionnaire included socio-demographic and medical information about the children, such as age, gender, parents’ education, developmental difficulties, health status, etc.

#### 2.2.2. The Behavior Rating Inventory of Executive Function—Parents Form (BRIEF) [56]

This standardized parent report, for children aged 5–18 years, characterizes their executive functions in natural, everyday environments [16]. The BRIEF consists of eight subscales: inhibit—resist impulses; shift—adjust allocation of attention and transition between tasks; emotional control—regulate and modulate emotion; initiate—start tasks; working memory—hold information in one’s immediate awareness long enough to perform a given task; plan/organize—use future orientation to complete steps in a sequence to meet a goal; organization of materials—effectively manage belongings; and monitor—self-check one’s progression with a task and adjust accordingly. The first three subscales combine to form the Behavioral Regulation Index (BRI), and the last five subscales combine to form the Metacognition Index (MI). The BRI and MI are combined into a Global Executive Composite (GEC) score. The BRIEF includes 86 statements describing various behaviors in which the parent rates the behavior frequency in the past six months on a Likert Scale ranging from 1 (never) to 3 (often). Higher scores on sub-scales, indices, and the GEC are indicative of more problems or difficulties with the executive function measured by the respective scale (score > 65). Test–retest reliability r = 0.86 for GEC, r = 0.88 for BRI, r = 0.84 for MI [56].

#### 2.2.3. The Virtual Action Planning Supermarket (VAP-S) [69]

The VAP-S is a shopping task applied in a virtual supermarket and assesses EFs as expressed in an IADL assignment. The individual is asked to purchase seven items from a list of products from various categories, such as milk products, fruits, and vegetables. The child should then proceed to the cashier’s desk and pay for the products. A training task that is similar but not identical to the test is also available to enable the user to become acquainted with the virtual environment. To select a product, the participant presses the left mouse button; the item automatically moves to the cart and its icon disappears from the displayed shopping list. When the participant is ready to pay, they must go to a checkout counter where a cashier is present. Otherwise, payment cannot be completed. At the cashier check-out counter, the participant places the items on the conveyor belt by pressing the left mouse button with the cursor pointing to the belt. The participant presses the left mouse button again to return the items to the cart. Payment is achieved by clicking on the wallet icon. The task is completed when the participant proceeds to the supermarket exit with the cart. While performing the task, visual and auditory distractions (such as other shoppers, signs or objects, and background sounds or music) appear to assess EFs, such as attention and inhibition [67,70]. The present study used the upgraded version of the virtual supermarket VAP-S 2 [67], which was visually improved and yielded more outcome measures. The VAP-S versions were modified to resemble an Israeli supermarket: the relevance of the products, the names of the aisles and grocery items, and all elements of the task were translated into Hebrew and Arabic [67,70]. The VAP-S was shown to be an ecologically valid assessment of EF in people with schizophrenia, Parkinson disease, people with mild cognitive impairment (MCI) and people with stroke [71,73].

**Figure 1:**
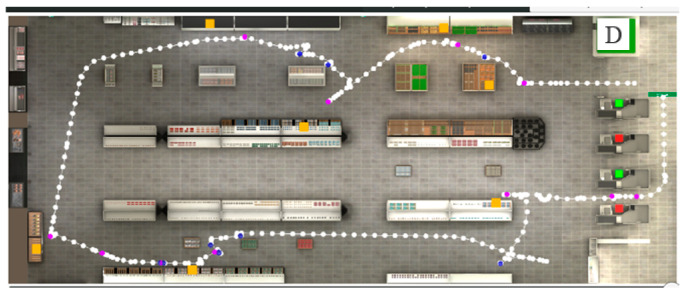
Picture showing the trajectory (path) of a participant during performance of the VAP-S. The departure of the path is indicated with the letter D. White dots correspond to the participant’s recorded positions. The orange squares represent the places where products appear on the shopping list. The purple dots represent participants’ stops. The blue dots represent collisions made by the participant. The green squares represent checkout counters with a cashier present. The red squares represent checkout counters without a cashier.

Outcome Measures: The VAP-S produced a variable report calculated from the recorded data. These included the total distance in meters traversed by the participant (referred to as the trajectory, Figure 1), the total time in seconds it took the user to complete the task, the number of items purchased (the number of items found and successfully moved into the cart), the number of correct actions (i.e., going to a checkout counter with an attending cashier and not to the one with no cashier; placing the items on the conveyor belt; removing the items from the conveyor belt; paying and exiting the supermarket), the number of incorrect actions (i.e., selecting items were not included in the list; selecting same item twice; going to a check-out counter without an attending cashier; exiting the supermarket without paying; staying within the super), the number of pauses, the combined duration of pauses in seconds, and the time to pay (i.e., the time between when the cost was displayed on the screen and when the participant clicked on the purse icon). In addition, The VAP-S automatically recorded the participant’s positions to provide an overview of the trajectory, collisions, and stops they made during the virtual shopping task (Figure 1). The eight outcomes can be conceptualized in terms of executive functioning into two categories: (1) variables that evaluate “task completion” as measured by number of purchased products and correct actions (2 variables); and (2) variables that evaluate “efficiency”, which is defined as competency in performance or ability to complete work with minimum expenditure of time and effort; efficiency was measured by time, distance, and incorrect actions (6 variables) [71]. To summarize, the main EF components were measured by looking at the participants’ planning abilities within the VAP-S and their organization in time and space [71].

#### 2.2.4. The Pediatric Quality of Life Inventory (PedsQL) [88]

The PedsQL assesses QoL in children and adolescents aged between 2 and 18 years. The PedsQL has a self-report version (for children above 5 years) as well as a parent-report version (used in the present study) and consists of 23 items that evaluate four domains of QoL: physical, emotional, social, and school-related QoL. The three domains of emotional, social, and school-related QoL comprise the total score for psychosocial QoL. In addition, the total QoL score is calculated for all the items. The questions are phrased in terms of the frequency of problems experienced over the past month used a 5-point Likert. The scores are converted into a scale of 0 to 100. A higher score indicates a higher QoL.

### 2.3. Procedure

After receiving permission from the Ethics Committee of the University of Haifa, Ministry of Education, advertisements for participating in the study were published in schools in the north of Israel, calling papers and children to participate in a study on EF and QoL among D/HH children. Parents who agreed to participate with their child in the study contacted the study conductor via phone call, in which they were asked several questions to ensure inclusion criteria. A meeting was set between the study conductor, parents, and children in the child’s school. In this meeting, the parents signed a consent form, and the children approved their consent to participate in this study. The parents completed the socio-demographic questionnaire, the BRIEF, and the PedsQL, while their children completed the VAP-S in a quiet room.

### 2.4. Data Analysis

All statistical analyses were conducted using the SPSS-25 program. Descriptive statistics were calculated for all measures. T test and chi-square tests were performed to examine the differences between the two groups in the relevant demographic variables. Normality tests were applied, and most of the dependent variables showed an abnormal distribution. Hence, the Mann–Whitney test was used to examine whether significant differences existed between the groups in BRIEF, VAP-S, and PedsQL scores. Among D/HH children, the Spearman test was used to examine the correlations between the outcome measures. Based on the correlation results, stepwise linear regression was used to test the contribution of BRIEF and VAP-S scores to the prediction of QoL. As predictors of the emotional, school-related, psychological, and total QoL domains, the following parameters were inserted into the regression: VAP-S: Total distance, total time, and efficiency scores, and BRIEF: MI, BRI, and GEC. To predict the physical QoL domain, the following parameters were inserted into the regression: VAP-S: Total distance score; BRIEF, MI, BRI, and GEC scores. For predictions of the social QOL domain, the BRIEF-MI, BRI, and GEC scores were only inserted into the regression. The correlation between mothers’ years of education, socioeconomic level, and the research variables was examined. The results revealed significant findings; thus, this variable was included as a step in the regression analysis. The level of significance was set at *p* ≤ 0.05 for all statistical tests.

## 3. Results

### 3.1. Hypothesis 1: Comparisons between Groups

When comparing EFs between D/HH and typical hearing children by BRIEF, the D/HH group scored significantly poorer in most BRIEF scales (which are indicated by higher scores)—inhibit, shift, emotional control, initiate, and working memory—in BRIEF-BRI and BRIEF total score (GEC). No significant differences were found in the BRIEF-MI score between the groups (Table 2).

When comparing EFs between groups, while performing the virtual shopping task (VAP-S), D/HH children scored significantly poorer in all outcome measures, except for ‘number of items purchased’, ‘number of correct actions’ and ‘time to pay’ (Table 3).

**Figure 2:**
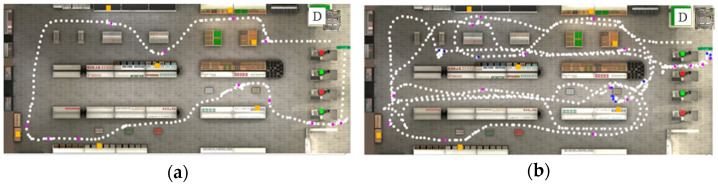
Trajectory (successive white dots) of participants during VAP-S performance. The departure of the path is indicated with the letter D. The orange squares represent the products, blue dots represent the collisions, and purple dots represent the stops. (**a**) Trajectory of a typical hearing child; (**b**) trajectory of a D/HH child. The green squares represent checkout counters with a cashier present. The red squares represent checkout counters without a cashier.

When comparing the trajectories of both groups in terms of time, distance, planning, etc., D/HH children showed less efficient performance: difficulties in planning and organization, for example, led to a longer trajectory, with more collisions and stops, and a higher number of incorrect actions (see examples in Figure 2).

When comparing QoL between groups as assessed by PedsQL, the D/HH group had significantly lower emotional, psychosocial, and school-related QoL, as reflected in the total PedsQL scores as well. No significant differences were found between the groups in physical and social QoL (Table 4).

### 3.2. Hypothesis 2: Examining the Correlations between BRIEF and VAP-S Scores, among D/HH Children

Among D/HH children, poorer EF, as measured by BRIEF, was correlated with lower performance in VAP-S. More significant correlations were found between VAP-S scores and BRIEF—emotional control, initiate, working memory, BRI and total score (GEC) (Table 5).

### 3.3. Hypothesis 3: Examining the Correlations between EF and QoL, among D/HH Children

Among D/HH children, lower EF, as measured by BRIEF and VAP-S, was correlated with lower QoL. More significant correlations were found with the emotional and school-related QoL (Table 6).

### 3.4. Hypothesis 4: Predicting QoL of D/HH Children by Their EF

Table 7 presents the results of Stepwise Linear regression analyses of all QoL domains. In general, VAP-S scores were not found to be significant predictors of QoL domains. When referring to QoL domains, the prediction of physical QoL yielded two models: the first included the BRIEF-MI score as a significant predictor, accounting for 44% of the variance (F = 33.3, *p* ≤ 0.001), and the second included the BRIEF-BRI score as a significant predictor, accounting for an additional 7% of the variance (F = 22.09, *p* ≤ 0.001). Emotional QoL was predicted by the BRIEF-GEC score, which accounted for 54% of the variance (F = 51.11, *p* ≤ 0.001). Social QoL was predicted by the BRIEF-BRI score, which accounted for 35% of the variance (F = 23.22, *p* ≤ 0.001). School-related QoL was predicted by BRIEF-MI score, accounting for 55% of the variance (F = 51.97, *p* ≤ 0.001). The prediction of psycho-logical QoL yielded one model in which the BRIEF-GEC score was a significant predictor, accounting for 65% of the variance (F = 80.81, *p* ≤ 0.001). The prediction of the total QoL score yielded one model in which the BRIEF-MI score was a significant predictor, accounting for 73% of the variance (F = 113.63, *p* ≤ 0.001).

## 4. Discussion

This is one of the first studies to examine the EF of D/HH children, as demonstrated by their everyday life settings according to parents’ reports and children’s daily activity performance in VR. This pilot study examined the differences in EF and QoL between D/HH children and typical hearing controls and explored the relationship between EF and children’s QoL among the study group. The main results were that D/HH children had significantly lower EF and QoL. Moreover, greater difficulties with EFs were related to a reduced QoL.

Based on the study results, the first hypothesis was confirmed. The differences in EFs between the groups were manifested in parents’ reports and in children’s actual activity performance in the virtual supermarket. These results support previous literature on EF difficulties in D/HH children [37,98,99]. Lin et al. [100] suggested that in D/HH children, greater numbers of neural resources are allocated for processing the degraded auditory incoming signals. This may create a cognitive load, limit available resources required for other processes, including EFs, and cause errors, information loss, and slower cognitive processing [101], all of which affect cognitive performance and daily function.

The present pilot study emphasizes the advantages of ecologically valid measures that reflect children’s EFs in daily life. According to the BRIEF, D/HH children had lower inhibition, shift, initiation, emotional control, and working memory scores, whereas on the plan/organize and monitor scales, the differences between the groups were not significant. Interestingly, Oberg and Lukomski [102], and Figueras et al. [30] obtained similar results. It is possible that plan/organize and monitor scales are less affected by hearing deprivation in D/HH children. Another possibility is that the timing of the development of various executive skills may place certain skills at greater risk during early school age, whereas others may not be affected until later ages. According to Garon et al. [103], executive skills such as inhibition and working memory may develop earlier than organizational skills, thus making the former more vulnerable than the latter during early school age [104]. Further studies on D/HH children are required to examine this point.

Gathering information from parents, as in BRIEF, is in line with the “family-centered” approach. This approach stresses the importance of including parents’ perspectives on their child’s challenges in daily life, in order to increase their awareness of the obstacles the child faces in daily life, improve parents’ and children’s involvement in therapy, and assist them in applying coping strategies and environmental adaptations to improve children’s daily function, development, and QoL [105]. However, parents’ reports are subjective and rely on their observations. Therefore, it is recommended that a child’s performance-based assessment be combined with the evaluation process [106].

The current study found significant differences in VAP-S scores between the groups. This result is supported by earlier studies that used VAP-S to evaluate EF; however, these previous studies were conducted on adult populations [16,54,69,71]. To the best of our knowledge, this is the first study to apply VAP-S in D/HH children. The results showed significant differences in the following VAP-S outcome measures: total distance, total time, number of incorrect actions, number of pauses, duration of pauses, and efficiency (sum of total time, total distance, and number of incorrect actions). These outcomes were found to discriminate between other clinical populations and normal controls with regard to EF as measured by the VAP-S, such as people with Parkinson’s disease, stroke, mild cognitive impairment, schizophrenia [16,54,69,71]. Nonetheless, the present pilot study is one of the first to demonstrate the feasibility of VAP-S in measuring EF in D/HH children. The differences in EF between D/HH children and typical hearing controls support VAP-S discriminant validity.

When referring to QoL, as hypothesized, D/HH children had significantly lower QoL than children with typical hearing. Differences were found in emotional QoL (which probably contributed to the difference in psychosocial QoL domains) and school-related QoL. These findings were similar to those reported by Rachakonda et al. [107] and Roland et al. [108]. However, this finding contrasts with that of Alnuhayer et al. [109], who found no significant difference in all PedsQL subscales between D/HH and typical hearing children aged 2–7 years old. In addition, no significant differences in physical QoL were found between the groups. It may be suggested that physical differences between D/HH children and their typical hearing peers at school age are smaller than the differences found in emotional and school-related QoL. The items included in both factors, such as worrying about the future, problems with paying attention in class, and missing school to go to a doctor or hospital, may be more significant in these ages.

The difference in social QoL between groups was close to being significant (*p* = 0.06). Further studies should verify whether this difference is significant by examining a larger sample of children. When referring to the significant differences between both groups in emotional and school-related QoL—psychosocial QoL—indeed, peer relationships and school functioning are increasingly becoming a focus of research investigating D/HH children. The present study supports previous reports highlighting the vulnerability of D/HH children to school and psychosocial functioning [107,108,110]. Parents of D/HH children frequently report their concern and wish that their children, albeit hearing loss, would be included in society, in school settings, and have good academic achievements [111,112], especially when the children study in a regular school system, as in the present study. Studies mention various reasons for the emotional load and its implications on emotional QoL of D/HH children: parents’ and teachers’ expectations, difficulties with expressive and receptive language development [113], difficulty in converting emotions into abstract concepts and verbal expression, distinguishing between the variety and intensity of emotions [114], as well as the growing social and academic demands and other functional restrictions, may explain the negative emotions such as anger and fear that children with cochlear implants experience [114,115].

Moreover, this pilot study found correlations between the emotional status of children in the study group (as demonstrated by the PedsQL) and their executive functions as measured by BRIEF and VAPS-2. EFs are a worthwhile domain to consider in relation to emotional states as they are also involved in a variety of psychopathologies [116]. From the perspective of developmental cognitive neuroscience, emotion and cognition are closely related and work together to process information and perform actions [117]. Bidirectional influences are likely to occur; cognitive processes play a part in regulating emotions, and emotions can assist people in organizing their thinking, learning, and behavior [118]. As mentioned in the literature, psychological distress may negatively affect executive functions among children in diverse populations like post-traumatic stress disorder (PTSD) [119], depression [120] and anxiety [121]. For instance, a recent systematic review and meta-analysis conducted by Nyvold et al. [119] indicated that children with PTSD have poorer executive functioning overall compared to normal controls. In addition, their findings showed that trauma and PTSD have a negative impact on multiple subdomains of executive function rather than just one or a few subdomains of EF. A similar result was reported in a depressed population, showing that children with higher levels of depressive symptoms had significantly impaired EF performance compared to normal controls [120]. Although not all children with depressive symptoms have an EF impairment, children who present with this comorbidity are at risk for additional cognitive impairments as well as significant psychiatric outcomes, such as longer hospitalizations [122]. Anxiety disorders have also been found to be associated with deficits in EF, as evidenced by studies that found that children with anxiety disorders performed lower than normal controls on some EF tasks [121,123]. Therefore, understanding how EFs are involved in the development of psychopathology may serve therapeutically useful purposes in both prevention and intervention [116].

Accordingly, the relationship between executive functions and emotional status should be further examined in D/HH children to better focus interventions on their specific needs. Researchers and clinicians should note the vicious cycle in which hearing loss leads to functional restrictions that may reduce children’s self-efficacy and enhance their emotional load [124,125]. Intervention programs should refer to the emotional profiles of D/HH children, provide help if needed, support children and parents, and enable them to cope better with threats and daily challenges.

When referring to the correlations between VAP-S and BRIEF scores. It is important to note that outcomes from several studies on the use of VR in the rehabilitation of executive functions, particularly VR for shopping, have been encouraging. The current study found significant correlations between the three outcome measures of VAP-S (total time, total distance, and efficiency) and varied BRIEF scores, indicating that these outcome measures appear to be components of EF. This is in line with the literature, which claims that complicated IADL requires the use of executive functions [126]. The significant correlations between the efficiency score of the VAP-S and the BRIEF-MI, BRI, and GEC scores suggest that the VAP-S measure necessitates planning, rule abiding, use of strategy, and organization in time and space, which are main EF components. These findings are consistent with earlier studies that found significant correlations between the VAP-S and the BADS for evaluating EF in people with schizophrenia and people with stroke [54,71].

In summary, the findings of this hypothesis support the use of the VAP-S as an ecologically valid measure of EF in D/HH children, although more studies are needed. Nonetheless, to deeply understand the obstacles and challenges that D/HH children face in their daily lives, it is essential to examine how health condition deficits, such as hearing loss and executive dysfunction, affect their ability to perform daily activities and participate in daily environments. Hence, in line with the ICF model [9], the present pilot study examined the relationship between EF and QoL in D/HH children. The results support the third and fourth hypotheses: greater deficits in EF are correlated with lower QoL, as measured in various domains. Knowledge of the relationship between EF and children’s QoL is limited. The existing literature in this regard is mainly focused on children with neurological conditions such as autism [127], epilepsy [128], and cerebral palsy [129], while studies on D/HH children are still lacking. The present study is one of the first to show the significant relationships between EF and physical, social, psychological, and school-related QoL in D/HH children by using ecological measures—parents’ report (BRIEF) and children’s actual daily activity performance (VAP-S). These results support reports on other clinical pediatric populations, such as children with epilepsy [128], sickle cell disease [130], and attention deficit hyperactivity disorder (ADHD) [131]. The findings highlight how difficulties in various EFs, such as working memory, the ability to regulate emotions, execute goal-directed behavior, and make decisions, may lead to poor coping and negatively affect almost all daily aspects, including academic achievements and social relationships [132,133], leading to cognitive and emotional load and reduced QoL. Given that EF has far-reaching effects on children’s cognitive, psychological, and social functions [134,135], EF evaluation should apply measures that reflect these various implications and their expressions in real life.

Interestingly, while correlations existed between the PedsQL domains and all BRIEF scales, only three VAP-S measures (total distance, total time, and efficiency) showed significant correlations with PedsQL domains. As mentioned above, the scores for total distance, total time, and efficiency are the main VAP-S scores derived from various EF components and are affected by low EF abilities. Hence, for example, a low efficiency score may reflect a lower ability to plan, organize, and monitor performance, a higher number of errors, longer time to complete the task, and longer trajectory. The fact that VAP-S successfully differentiated between the EF of D/HH children and typical hearing controls, similar to BRIEF, reflects the parallel validity of these measures.

However, BRIEF scores (MI, BRI, and GEC) were the strongest predictors of low QoL in all the measured domains, whereas VAP-S scores did not significantly predict children’s QoL. Therefore, further studies should examine the implications of EF deficits on the daily life of D/HH children, to elucidate functional aspects and reveal feasible measures that reflect them. This approach may assist in optimizing intervention programs for D/HH children and improve not only functions related to comorbidities such as EF but also enable children to better cope with real life in their natural environment. Thus, we may expect optimal development, child inclusion, and well-being.

The results of the present pilot study have important theoretical and practical implications. From a theoretical perspective, the results implement and broaden the ICF model in D/HH children by indicating that an association between body functions (i.e., EFs), activity performance, and QoL exists among these populations. The present study contributes to a deeper understanding of both the concepts of EF and QoL, which in turn contributes to the theoretical foundation of the ecological approach and the establishment of a biopsychosocial model of health. Furthermore, the findings highlight the EF challenges that D/HH children face in their daily life and explore the relationships between EF and QoL, which are still less studied among D/HH children, especially those from regular education.

These findings have practical and clinical implications beyond their theoretical contributions to the existing literature. From a clinical perspective, a deeper and more comprehensive understanding of EF difficulties in D/HH children and their consequences in daily life settings, together with the ability to understand the association between certain EF abilities and specific domains of QoL, may help clinicians implement direct, precise, and effective intervention processes. This helps parents to deal more effectively with the performance difficulties and challenges faced by their D/HH children in daily life. Increased awareness by parents and clinicians, as well as specific knowledge regarding the EF and QoL challenges faced by D/HH children in various settings, may contribute to evaluation and intervention processes that aim to promote their participation and quality of life.

Finally, the research supports receiving knowledge sources that come from both ecological tools based on the child’s performance in the context of real life, and from information from standard tools obtained from the parent’s point of view, which provides a different perspective on the child’s functioning in everyday life. This is undoubtedly an important issue and of great significance in the development of future evaluation and intervention programs.

## 5. Research Limitations

The current pilot study has several limitations. First, as a pilot study, it has a relatively small sample size, limiting the generalization of the results and conclusions. However, based on the findings of the present study, further studies on larger sample sizes should be performed to strengthen the current results and enable their generalization. Second, the study group included children that study in regular education schools, most of them had mild-moderate hearing loss, and thus may have better participation and inclusion, or better EF as compared to children with severe hearing impairment who study in special education. Hence, this may restrict the generalizability of the study results.

Additionally, in this study, most participants had bilateral hearing loss, but some had unilateral hearing loss; therefore, this study may not adequately represent the experiences of the broader population of D/HH children. It may be assumed that in each group, children may face unique challenges such as sound localization [136], social integration [137] and communication strategies [138]. Future studies should explore the specific experiences and needs of children with unilateral versus bilateral hearing loss to better understand the impact of hearing loss on their development and well-being.

Moreover, the present study relied solely on parent ratings of QoL. Additional studies with larger samples of children with diverse severities of hearing loss are recommended. These studies should apply ecological measures to elucidate deficits in EF, their relationship to hearing impairment severity, and QoL. The diversity of D/HH children will enable a better understanding of how their challenges affect their inclusion and development. These studies should use parents’ and children’s reports and examine whether any differences exist between them. Interventions should also use frameworks such as the ICF to bridge between hearing impairment, EF, and children’s function in real life. Another limitation is that the current study did not address the vestibular function of the study group and did not examine the effect of vestibular deficits on executive functions on EF. This emphasizes the necessity for further research into understanding the complex interaction between vestibular function and cognitive processes.

## 6. Conclusions

The current pilot study may provide a valuable contribution to the literature by outlining D/HH children’s profiles in terms of EF and QoL, as well as emphasizing and elucidating the association between EF and QoL in D/HH children. In this pilot study, several differences were found in the EF and QoL between D/HH and typical hearing controls, highlighting the relevance of screening EF in children with D/HH. The results revealed that EF deficits in D/HH children may be present in their daily lives and affect their activity performance and QoL. In addition, the importance of assessing EF using various tools has been highlighted in the present study. Ecological measures that reflect daily scenarios may help in assessing EF in D/HH children and in creating optimal interventions and training programs for parents and educators, to provide them with practical resources to use when working with D/HH children. These resources could encourage children to participate in more EF-promoting activities at home, in the community, and in school. In line with the ICF model, the children’s participation in EF activities may improve and enhance children’s daily function, QoL, and optimal development. Finally, investigating the relationship between EF and QoL may contribute significantly to the existing theoretical knowledge of these connections.

Notably, this pilot study is one of the first to examine executive functions through an ecological assessment, integrating both parent reports and the child’s performance in a virtual task. Notably, it is the first time that the VAP-S has been utilized with a population of children with hearing impairments. The research introduces professionals to the relevance of using a virtual platform in the assessment and treatment of children with hearing impairments, emphasizing the tool’s strengths and its sensitivity in identifying executive function difficulties within this population in a child-friendly manner.

## Figures and Tables

**Table 1:** Participants’ socio- demographic information and the study group’ hearing impairment characteristics.

	D/HH Children (*n* = 38)	Typical Hearing Controls (*n* = 38)	*t* (74)
	*M* ± *SD*	*M* ± *SD*	
Age	9.09 ± 1.37	9.41 ± 1.43	0.99
Mothers’ years of education	11.92 ± 2.48	16.05 ± 3.00	6.54 **
	Number (%)	Number (%)	χ^2^
Gender			0.84
Male	20 (52.6%)	16 (42.1%)	
Female	18 (47.7%)	22 (57.9%)	
Socio-economic level			16.13 **
Low	8 (21.1%)	4 (10.5%)	
Average	24 (63.2%)	11 (28.9%)	
High	6 (15.8%)	23 (60.5%)	
The study group’ hearing impairment characteristics			
Side of hearing impairment			
Unilateral	11 (28.9%)		
Bilateral	27 (71.1%)		
Severity of hearing impairment			
Mild (20–35 dB)	13 (34.2%)		
Moderate (35–50 dB)	21 (55.3%)		
Moderately- severe (50–65 dB)	2 (5.3%)		
Severe (65–80 dB)	2 (5.3%)		
Type of hearing loss			
Conductive	11 (28.9%)		
Sensorineural	26 (68.4%)		
Mixed	1 (2.6%)		
Device used			
Hearing aid	30 (78.9%)		
Cochlear implant	3 (7.9%)		
Hearing aid + Cochlear implant	5 (13.2%)		
Mode of communication			
Oral	37 (97.4%)		
Signing	0		
Oral + Signing	1 (2.6%)		

** *p* ≤ 0.01. M = mean; SD = standard deviation. The socio-economic level was defined according to the criteria published by the Israeli statistical bureau (2020). The severity of hearing impairment was classified according to the World Health Organization criteria [1].

**Table 2:** The differences between groups in BRIEF scores.

	D/HH Children (*n* = 38)	Typical Hearing Controls (*n* = 38)	*Z*
	*Md (IQR)*	*Md (IQR)*	
Inhibition	47 (8)	42 (7)	3.79 **
Shift	43 (11.75)	49 (5.25)	2.11 *
Emotional control	45 (7.25)	40 (5.25)	3.19 **
Initiate	43 (11.75)	40 (8)	2.41 *
Working memory	44 (8.75)	40 (6.25)	1.93 *
Plan/Organize	38 (11.25)	38 (3)	0.68
Organization of materials	39 (8.25)	37 (9)	0.17
Monitor	35 (6.5)	35 (5.5)	0.88
BRI	44 (11)	40 (5.25)	3.66 **
MI	38 (10.25)	37 (5.5)	0.81
GEC	45 (12)	43 (5.5)	2.19 *

* *p* ≤ 0.05, ** *p* ≤ 0.01. Md = Median; IQR = Inter quartile range. BRI = Behavioral Regulation Index; MI = Metacognition Index; GEC = Global Executive Composite.

**Table 3:** The differences between groups in VAP-S scores.

	D/HH Children (*n* = 38)	Typical Hearing Controls (*n* = 38)	*Z*
	*Md (IQR)*	*Md (IQR)*	
Total distance (meters)	190.91 (82.22)	147.78 (32.33)	3.83 **
Total time (seconds)	581.81 (203.17)	420.61 (122.96)	4.57 **
Number of items purchased	7 (0)	7 (0)	0.00
Number of correct actions	13.00 (0)	13.00 (0)	1.00
Number of incorrect actions	6.50 (9.5)	4.00 (4.25)	2.72 **
Number of pauses	14.00 (8.25)	10.50 (4.5)	2.68 **
Duration of pauses (seconds)	196.31 (132.8)	135.22 (80.88)	3.41 **
Time to pay	6.14 (5.91)	5.34 (5.09)	0.88
task completion	20 (0)	20 (0)	1.00
Efficiency	792.09 (269.51)	564.02 (134.12)	4.74 **

** *p* ≤ 0.01. Md = Median; IQR = Interquartile range.

**Table 4:** The differences between groups in PedsQL scores.

	D/HH Children (*n* = 38)	Typical Hearing Controls (*n* = 38)	*Z*
	*MD (IQR)*	*MD (IQR)*	
Physical QoL	100 (12.5)	100 (12.5)	0.06
Emotional QoL	90 (25)	100 (15)	1.96 *
Social QoL	100 (14.06)	100 (1.56)	1.34
School QoL	80 (21.25)	100 (10)	4.02 **
Psychosocial QoL	90 (19.37)	96.67 (8.75)	3.41 **
Total QoL	92.5 (16.09)	96.56 (7.62)	3.43 **

* *p* ≤ 0.05, ** *p* ≤ 0.01. Md = Median; IQR = Inter quartile range.

**Table 5:** The significant correlations between BRIEF and VAP-S scores among D/HH children.

	*VAP-S*			
		Total Time	Total Distance	Efficiency
*BRIEF*	Inh	NS	0.31 **	0.20 *
	Sh	NS	0.40 **	0.25 *
	EmC	0.38 **	0.48 **	0.43 *
	Init	0.21 *	0.40 **	0.28 **
	WM	0.25 *	0.36 **	0.30 *
	P/O	NS	0.20 *	NS
	OoM	NS	0.19 *	NS
	Mo	NS	NS	NS
	BRI	0.28 **	0.46 **	0.35 **
	MI	NS	0.32 **	0.20 *
	GEC	0.20 *	0.40 **	0.26 *

NS: not significant; * *p* ≤ 0.05, ** *p* ≤ 0.01. Only significant correlations are presented in the table. Variables that did not significantly correlate were removed from the table. Inh = Inhibition; Sh = Shift, EmC = Emotional control; Init = Initiate; WM = Working memory; P/O = Plan/Organize; OoM = Organization of materials; Mo = Monitor; BRI = Behavioral Regulation Index; MI = Metacognition Index; GEC = Global Executive Composite.

**Table 6:** The significant correlations between BRIEF, VAP-S and PedsQL scores among D/HH children.

	PedsQL						
		Physical QoL	Emotional QoL	Social QoL	School QoL	Psychosocial QoL	Total QoL
*VAP-S*	Total distance	−0.30 *	−0.37 *	NS	−0.40 **	−0.30 *	−0.31 *
	Total time	NS	−0.39 **	NS	−0.30 *	−0.31 *	−0.29 *
	Efficiency	NS	−0.44 **	NS	−0.34 *	−0.35 *	−0.34 *
*BRIEF*	Inh	NS	NS	−0.24 *	−0.38 **	−0.31 **	−0.30 **
	Sh	−0.20 *	−0.59 **	−0.57 **	−0.51 **	−0.63 **	−0.56 **
	EmC	−0.27 **	−0.52 **	−0.47 **	−0.47 **	−0.55 **	−0.52 **
	Init	−0.42 **	−0.44 **	−0.45 **	−0.63 **	−0.60 **	−0.60 **
	WM	−0.34 **	−0.52 **	−0.30 **	−0.63 **	−0.58 **	−0.58 **
	P/O	−0.48 **	−0.47 **	−0.43 **	−0.33 **	−0.39 **	−0.41 **
	OoM	NS	NS	NS	−0.29 **	−0.21 *	−0.21 *
	Mo	−0.42 **	−0.29 **	−0.33 **	−0.24 *	−0.25 *	−0.28 **
	BRI	−0.26 *	−0.48 **	−0.49 **	−0.52 **	−0.56 **	−0.52 **
	MI	−0.44 **	−0.50 **	−0.41 **	−0.52 **	−0.51 **	−0.52 **
	GEC	−0.39 **	−0.51 **	−0.46 **	−0.58 **	−0.56 **	−0.55 **

NS: not significant; * *p* ≤ 0.05, ** *p* ≤ 0.01. Only significant correlations are presented in the table. Variables that did not significantly correlate were removed from the table. Inh = Inhibition; Sh = Shift, EmC = Emotional control; Init = Initiate; WM = Working memory; P/O = Plan/Organize; OoM = Organization of materials; Mo = Monitor; BRI = Behavioral Regulation Index; MI = Metacognition Index; GEC= Global Executive Composite.

**Table 7:** Predicting quality of life among D/HH children by BRIEF scores.

	Model 1			Model 2		
	*B*	SE *B*	*β*	*B*	SE *B*	*β*
Physical QoL						
BRIEF−MI	−0.82	0.14	−0.66 ***	−1.26	0.22	−1.01 ***
BRIEF−BRI				0.73	0.28	0.45 *
R^2^		0.44			0.51	
F for change in R^2^		33.3 ***			6.56 *	
Emotional QoL						
BRIEF−GEC	−1.29	0.18	7.74 ***			
R^2^		0.54				
F for change in R^2^		51.11 ***				
Social QoL						
BRIEF−BRI	−1.17	0.24	−0.59 ***			
R^2^		0.35				
F for change in R^2^		23.22 ***				
School QoL						
BRIEF−MI	−1.01	0.14	−0.74 ***			
R^2^		0.55				
F for change in R^2^		51.97 ***				
Psychosocial QoL						
BRIEF−GEC	−1.12	0.13	−0.81 ***			
R^2^		0.65				
F for change in R^2^		80.81 ***				
Total QoL						
BRIEF−MI	−0.94	0.09	−0.85 ***			
R^2^		0.73				
F for change in R^2^		113.63 ***				

* *p* ≤ 0.05, *** *p* ≤ 0.001. BRI = Behavioral Regulation Index; MI = Metacognition Index; GEC = Global Executive Composite.

## Data Availability

Data are unavailable due to privacy and ethical restrictions.
